# Bos taurus genome sequence reveals the assortment of immunoglobulin and surrogate light chain genes in domestic cattle

**DOI:** 10.1186/1471-2172-10-22

**Published:** 2009-04-30

**Authors:** Anna Ekman, Mikael Niku, Jenni Liljavirta, Antti Iivanainen

**Affiliations:** 1 Department of Basic Veterinary Sciences, University of Helsinki, Helsinki, Finland; 2 Department of Applied Chemistry and Microbiology, University of Helsinki, Helsinki, Finland

## Abstract

**Background:**

The assortment of cattle immunoglobulin and surrogate light chain genes has been extracted from the version 3.1 of *Bos taurus *genome sequence as a part of an international effort to sequence and annotate the bovine genome.

**Results:**

63 variable lambda chain and 22 variable kappa chain genes were identified and phylogenetically assigned to 8 and 4 subgroups, respectively. The specified phylogenetic relationships are compatible with the established ruminant light chain variable gene families or subgroups. Because of gaps and uncertainties in the assembled genome sequence, the number of genes might change in the future versions of the genome sequence. In addition, three bovine surrogate light chain genes were identified. The corresponding cDNAs were cloned and the expression of the surrogate light chain genes was demonstrated from fetal material.

**Conclusion:**

The bovine kappa gene locus is compact and simple which may reflect the preferential use of the lambda chain in cattle. The relative orientation of variable and joining genes in both loci are consistent with a deletion mechanism in VJ joining. The orientation of some variable genes cannot be determined from the data available. The number of functional variable genes is moderate when compared to man or mouse. Thus, post-recombinatorial mechanisms might contribute to the generation of the bovine pre-immune antibody repertoire. The heavy chains probably contribute more to recombinational immunoglobulin repertoire diversity than the light chains but the heavy chain locus could not be annotated from the version 3.1 of *Bos taurus *genome.

## Background

Immunoglobulins are the molecular mediators of the adaptive humoral immune response in jawed vertebrates. Somatic recombination during B lymphoid differentiation is required for immunoglobulin expression [1]. In the germline state, the genes encoding for the variable (V), diversity (D) and joining (J) segments are dispersed across a wide genomic stretch. A process called V(D)J joining brings together the specific genes for each segment type and thereby creates the second exon of a transcriptionally competent immunoglobulin gene. The recombination machinery consists of two recombination activating gene products RAG1 and RAG2 as well as various other proteins, reviewed in [2]. The *cis*-acting recognition signal sequences (RSSs) target the recombination machinery to the correct genomic site. Each RSS consists of heptamer and nonamer motifs flanking a 12 or 23 bp long central spacer. In the rearranging locus, two variably separated double strand DNA breaks are introduced next to one 12 bp and one 23 bp RSS. The nascent non-homologous DNA ends are joined into a coding joint in the middle of the recombined gene. The DNA fragment between the breaks is either deleted or inverted depending on the relative orientation of the recombining genes.

The immunoglobulin heavy chain and light chain rearrangements in many species are temporally separated during B cell development. In mouse and man but not in chicken, a population of cells can be demonstrated that has undergone rearrangement only in the immunoglobulin heavy chain locus [3,4]. A surrogate light chain (SLC) is temporarily expressed at this stage of the B cell development [5]. SLC is composed of two polypeptides VPREB and IGLL1 that are homologous to the variable and the constant domain of the immunoglobulin light chain, respectively [6]. In mice, three VPREB paralogues *VPREB1*, *VPREB2 *and *VPREB3 *have been described [7,8]. The IGLV-like *VPREB2 *is missing from the human genome. Gene targeting studies demonstrate the role of SLC genes in the production of B cells [9].

The genome sequence of *Bos taurus *permits for the first time a direct estimate of the size of the immunoglobulin light chain gene pool in domestic cattle, one of the most important farm animal species. We have characterized the structure and composition of bovine immunoglobulin and surrogate light chain gene loci as a part of a community effort to annotate the version 3.1 assembly of *Bos taurus *genome sequence [10].

## Results

The bovine immunoglobulin lambda (λ) chain locus is located on chromosome 17. In version 3.1 of the genome sequence (Btau_3.1), 63 variable, 3 joining and 5 constant genes could be identified in 10 scaffolds. 25 λ variable genes (ca. 41%) fulfilled the criteria for classification as potentially functional (see Methods and Additional file 1).

**Figure 1:**
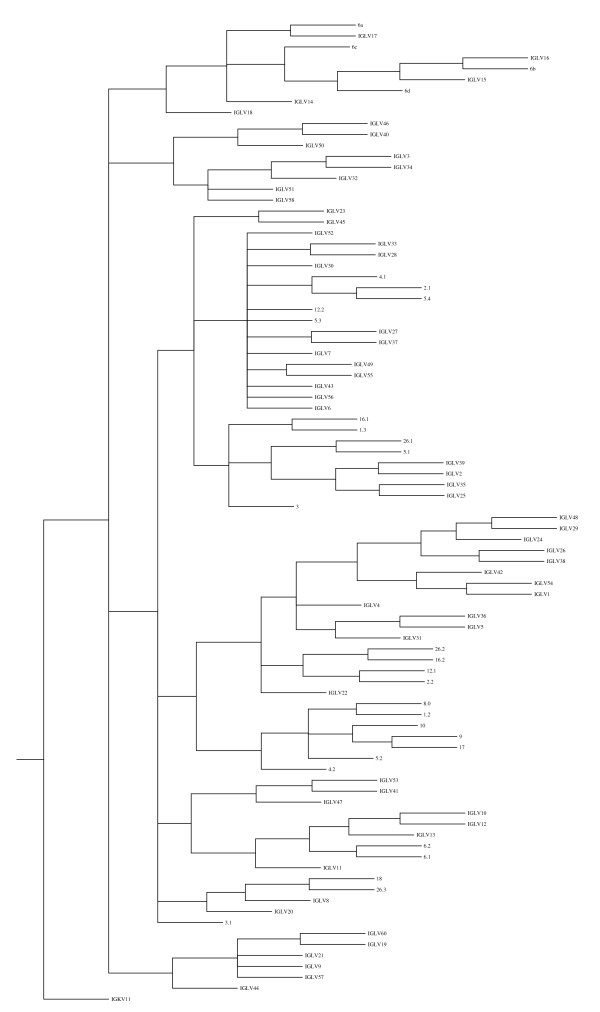
**Phenogram of ruminant immunoglobulin λ variable genes**. Sequences corresponding to the V region but excluding CRD3 were aligned and phylogenetically analyzed as described in the methods. *IGKV11 *was used as an outgroup. Ovine genomic sequences 1.2, 1.3, 2.1, 2.2, 3, 3.1, 4.1, 4.2, 5.1, 5.2, 5.3, 5.4, 6.1, 6.2, 8.0, 9, 10, 12.1, 12.2, 16.1, 16.2, 17, 18, 26.1, 26.2, 26.3 (AF040900–AF040924, M60441) are from Reynaud *et al*. [13] Ovine sequences 6a, 6b, 6c and 6d (AF038145–AF038148) are derived from cDNA [14].

Based on the phylogenetic analyses and nucleotide sequence identities in a gene region corresponding to FR1–FR3, the λ variable genes can be grouped into 8 phylogenic subgroups (figure 1, Additional files 1 and 2). The λ variable gene subgroups in the present work accommodate all the characterized bovine *IGLV *genes from [11] and most of the ovine *IGLV *genes [12-15]. Interspecies comparison revealed that four of the six described ovine gene families or subgroups [12-15] are represented in the bovine collection (figure 1 and Additional file 2) and contain 43 (ca. 68%) of the bovine genes. As can be seen from Additional file 1, subgroup 1 is the largest and contains 16 (ca 64%) of the potentially functional λ variable genes. This subgroup seems to be ruminant specific as no human or mouse genes co-segregate with its members. Subgroups 2 and 6 are each represented in the genome by a single subgroup-specific gene cluster. The 13 bovine genes of subgroup 5 are all pseudogenes as are the ovine genes in this subgroup. With the exception of one gene, the bovine (but not the ovine) genes in this subgroup share an in-frame stop codon in framework 3 (not shown). 20 genes (ca. 32%) of which 3 are potentially functional do not co-segregate with any members of the established ovine λ variable gene subgroups. However, λ variable genes in the bovine subgroups 7, 8 and 9 are similar to genes in human specific subgroups 5, 8 and 9 respectively (i.e., 80% nucleotide sequence identity in a gene region corresponding to FR1–FR3; not shown). No ovine, human or mouse genes closely related to *IGLV41*,*IGLV47 *or *IGLV53 *could be identified. Further, no bovine genes from the current assembly could be mapped to the established ovine families III or IV [12-15].

Three immunoglobulin lambda joining and five immunoglobulin lambda constant genes were identified (Additional file 1). Two of the J-C gene pairs form apparently functional units. *IGLC1 *and *IGLC2 *have identical coding sequence but differ at 3'UTR. Chen *et al*. [16] described four *IGLC *genes which correspond to *IGLC2-IGLC5 *in this paper.

**Figure 2:**
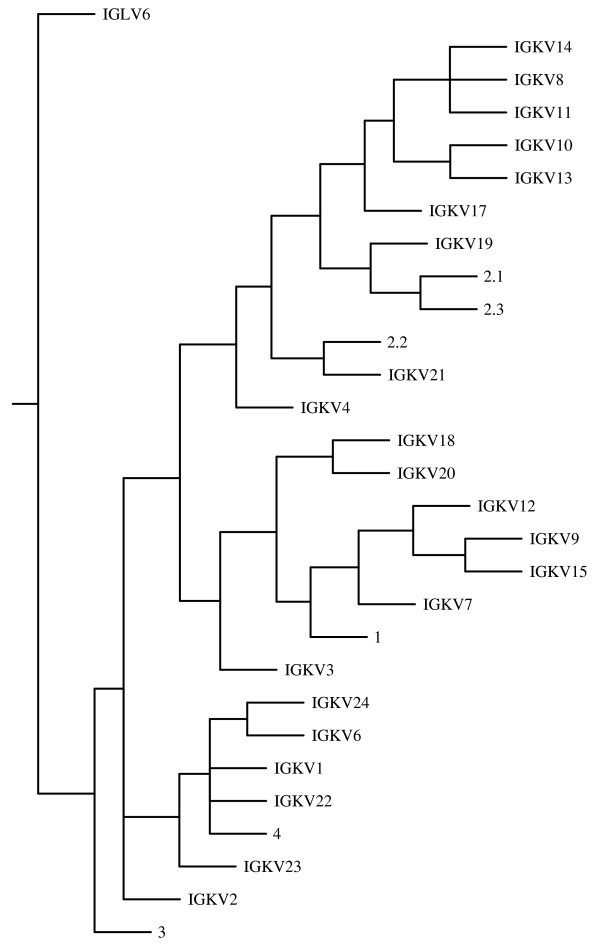
**Phenogram of ruminant immunoglobulin κ variable genes**. Sequences corresponding to the V region but excluding CRD3 were aligned and phylogenetically analyzed as described in the methods. *IGLV6 *was used as an outgroup. The ovine sequences 1, 2.1, 2.2, 2.3, 3 and 4 (AF038133–AF038138) are derived from cDNA [14].

The bovine immunoglobulin κ locus is located in chromosome 11. A blast search against Btau_3.1 revealed matches only at a single location in scaffold Chr11.003.53. 22 variable, 3 joining and one constant immunoglobulin κ gene were identified. 8 variable genes (ca. 36%) were classified as potentially functional (Additional file 3). The variable κ genes can be grouped in 4 phylogenic subgroups preserving the established ovine specific gene families (figure 2 and Additional file 4). 21 (ca. 95%) of the bovine genes co-segregate with ovine κ variable gene families I, II and IV [15]. Subgroup 2 is the largest and contains 7 (ca. 88%) of the potentially functional genes. All the seven members of subgroup 1 are pseudogenes.

**Figure 3:**
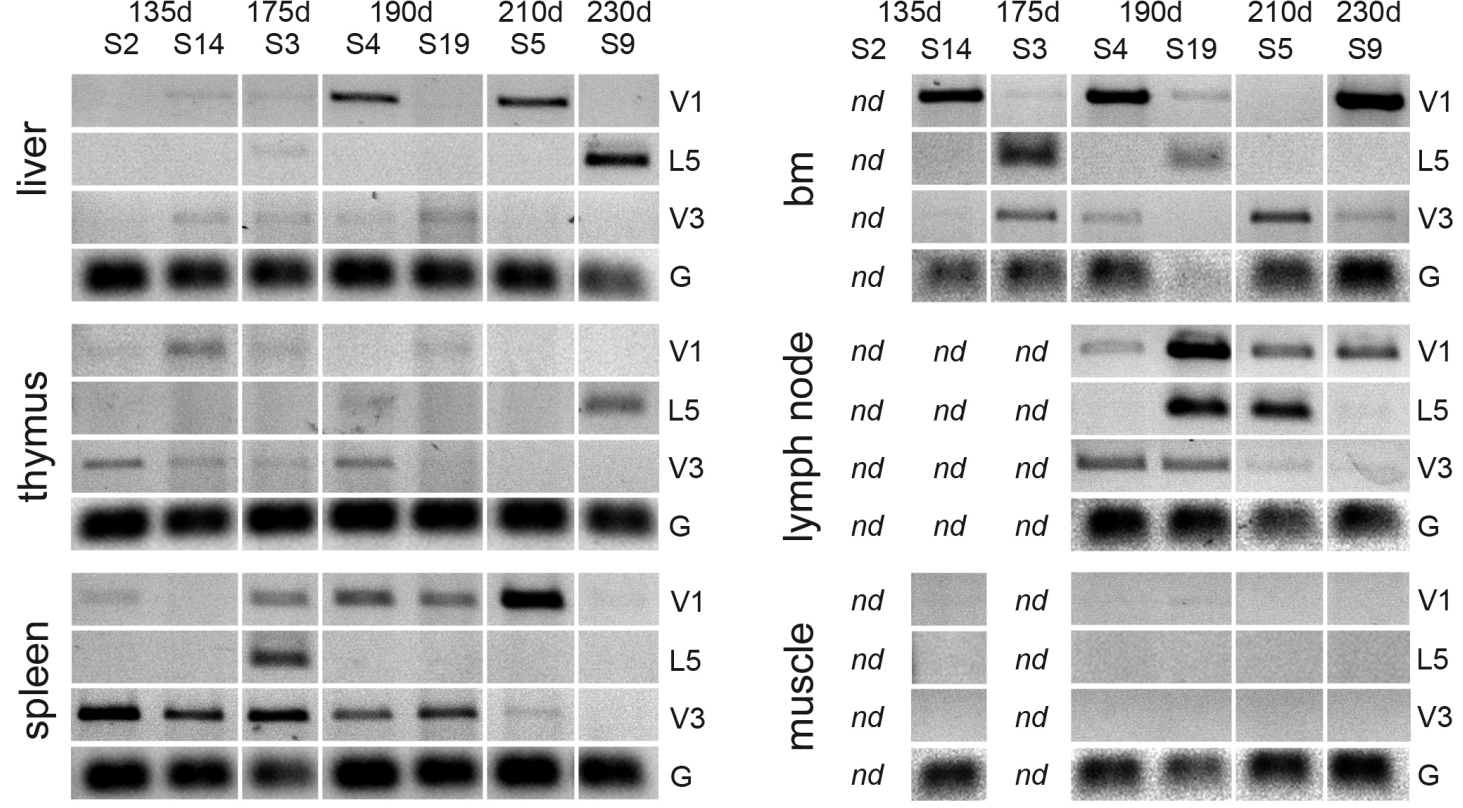
**Expression of VPREB1, VPREB3, and IGLL1 in bovine fetuses**. Gene expression was analyzed by RT-PCR as described in methods. The individual animals and their estimated fetal ages are indicated on top. The amplicon lengths were: 558 bp (*VPREB1*), 491 bp (*VPREB3*), 475 bp (*IGLL1*) and 150 bp (*GAPDH*). V1 = *VPREB1*, V3 = *VPREB3*, L5 = *IGLL1*, G = *GAPDH*, bm = bone marrow, nd = not determined.

Surrogate light chain genes *VPREB1*, *IGLL1 *and *VPREB3 *were identified in the assembly at or close to the λ chain locus. The *VPREB1 *and *IGLL1 *lie next to each other as in the mouse genome [8]. *VPREB3 *is located close to the immunoglobulin λ constant genes *IGLC1-IGLC4 *but in the opposite transcriptional orientation. None of the surrogate light chain genes is flanked by an RSS. The exon-intron boundaries of the surrogate light chain genes are conserved between cow, mouse and man (not shown). The *VPREB1 *and *VPREB3 *gene structures resemble those of the immunoglobulin λ variable genes with a leader and main exon. Successful cloning of the cDNAs using primers that span the exon/intron boundaries and extend far into the 3'UTR of the germline genes confirms that the mRNA expression of surrogate light chain genes does not depend on recombination. The functionality of the surrogate light chain genes was additionally supported by demonstrating the expression of *VPREB1*, *VPREB3 *and *IGLL1 *mRNA in fetal liver, spleen, bone marrow, lymph node and thymus (figure 3).

The cDNA and genomic DNA sequence analysis of the surrogate light chain genes revealed several single nucleotide differences in comparison with the reference genomic sequence (Additional file 5). Therefore, it seems that the bovine surrogate light chain genes are polymorphic.

## Discussion

In this paper, we have presented the analysis of the immunoglobulin and surrogate light chain gene assortment extracted from the *Bos taurus *genome sequence Btau_3.1 [10]. Btau_3.1 is nearly completely based on a whole genome shotgun sequence from a single animal (L1 Dominette 01449) with a 30% inbreeding coefficient [10,17]. This facilitates the analysis of immunoglobulin genes, which is in mixed databases greatly complicated by gene polymorphism and targeted somatic mutations [18]. Most of the functional light chain genes have probably been included in our gene set although the exact number of genes is likely to change in the future genome versions. 32 λ variable genes were in genomic contigs not assigned to a specific chromosomal location and might include orphons.

**Figure 4:**
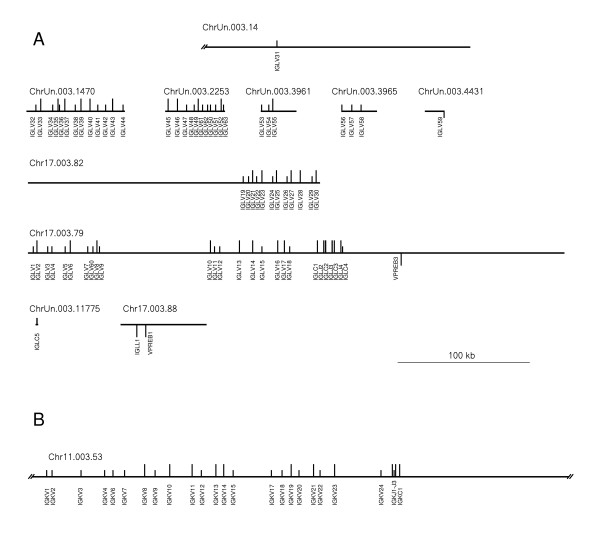
**Schematic organization of the kappa, lambda and surrogate light chain genes in Btau_3.1 scaffolds**. The genes on the plus strand are projected above and those on minus strand below the scaffold line. Long projection stands for an apparently functional gene and a short projection for a nonfunctional gene. Scale bar: 100 kb. A: Lambda and surrogate light chain genes. B: Kappa light chain genes.

An interspecies comparison suggests ruminant specific adaptations:

(1.) The bovine κ locus appears small and uncomplicated when compared with the λ locus (see figure 4). This might reflect the preferential use of the λ light chain in cattle [19].

(2.) In cattle, the number of functional immunoglobulin light chain genes is markedly lower than in mice and in man, i.e. 33 vs. 105 and 77 [20]. Further, the variation in CDR1 length is more restricted and the number of unique CDR1–CDR2 combinations is lower than in mice and man (see table 1). Even though some genes might be missing from Btau_3.1, the number of potentially functional bovine light chain genes probably overestimates the *bona fide *functional genes for which protein evidence is required. We are not aware of any other approximations on the number of functional immunoglobulin genes in ruminants. By extrapolation, the total number of λ variable gene segments in the sheep genome has been estimated from 60–90 [14] to 150 [18]. The latter estimate is, however, based on cDNA data.

**Table 1:** Characteristics of CDR1 and CDR2 in the variable regions of bovine, mouse and human light chains

	**cattle**	**mouse**	**man**
**lambda chain variable region**			
CDR1 length (amino acids)	6,8,9	7,8,9	6,7,8,9
CDR2 length (amino acids)	3,7	3,7	3,7
unique IGLV CDR1/CDR2 pairs	19	8	37

**kappa chain variable region**			
CDR1 length (amino acids)	6,10,11	5,6,7,10,11,12	6,7,10,11,12
CDR2 length (amino acids)	3	3	3
unique IGKV CDR1/CDR2 pairs	5	94	27

The data on human and murine immunoglobulins are from IMGT database [20].

(3.) The phylogenetic analysis suggests that most of the potentially functional λ genes belong to a single subgroup (subgroup 1, see additional file 6) that is not apparent in the human or in the mouse genomes but is present in sheep genome. This subgroup comprises 21 variable genes of which 16 are potentially functional. The CDR1 [21] is either 8 or 9 amino acids long with a characteristic hydrophobic residue at position 30. Based on similarities on primary sequences, the CDR 1 structures among the members of subgroup 1 correspond most closely to the canonical loop 1 structures 1 and 2 found on λ chain variable regions [22]. CDR2 is 3 amino acids long and probably adopts a hair pin structure commonly found on CDR2 of λ and κ light chains [22]. It remains to be seen whether or not the CDRs adopt any of the established canonical immunoglobulin structures in reality. No high resolution structures are currently available for bovine immunoglobulins in the PDB archives [23].

(4.) The apparent expansion of the pseudogene subgroup 5 is intriguing although the reasons behind this are currently elusive. 12 subgroup members out of 13 share an identical stop codon in framework 3.

The data on the overall organization of the bovine λ chain locus is still quite fragmentary (figure 4). It could resemble the human locus, which displays a 900 kb long upstream region of 73 to 74 variable genes followed by 7 to 11 pairs of joining and constant genes all in one transcriptional orientation [24]. However, recombination using inversion cannot be ruled out in the bovine λ chain locus at present. In contrast to what is found in man and cattle, the murine λ chain locus is much reduced in size (only about 240 kb) and contains two small clusters of different immunoglobulin lambda chain genes [reviewed in [25]].

**Figure 5:**
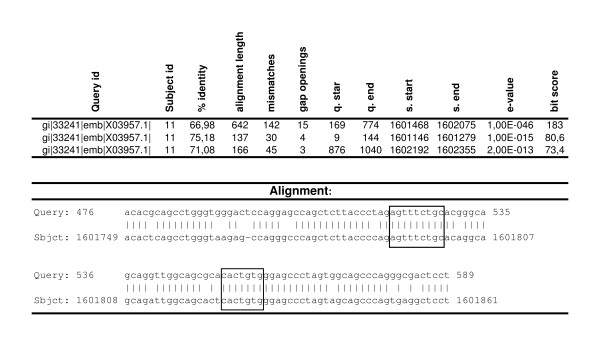
**Pair wise alignment of the human kappa deleting element [gi = 33241] and Chr11.003.59**. Upper part: tabular output from bl2seq. The following parameters were used: blastn matrix: 2, -3; gap open: 5, gap extension: 2. Lower part: local alignment about 28.5 kb downstream from IGKC1. Heptanucleotide and nonanucleotide boxes are emphasized.

The κ chain locus is much less complicated in cattle than in man or mouse. All identified κ genes were localized to a ca. 280 kb genomic segment within Chr11.003.53 (Additional file 3 and figure 4). In comparison, the κ locus spans ca. 1.8 Mb in man [26] and ca. 3.2 Mb in mouse [27,28]. The relative orientation of the bovine genes allows recombination exclusively by deletion. In mice, the relative orientation of more than 75 of the 140 murine κ variable genes supports inversion [29,30]. In man, a large duplication has produced nearly identical copies of 34 variable κ genes ca. 800 kb upstream in an opposite orientation. In addition, 2 variable genes most proximal to the J-C gene region support inversion [26]. A kappa deleting element (kde) homologous to the murine recombining sequence (RS) is located about 24 kb downstream of the human κ locus [31]. A permanent disruption of one or both κ loci by a recombination involving kde (RS) is a frequent finding in human and murine B cells that produce the λ light chain [32,33]. Interestingly, a highly similar sequence to kde is located 28.5 kb downstream of the bovine κ locus (see figure 5).

The heavy chain locus could not be annotated as most of it is missing from Btau_3.1. The available data on the light chain loci suggests that a moderate number of potentially functional light chain genes exist in the bovine genome. Although the heavy chains add more to the recombinatorial diversity of immunoglobulins than the light chains, post-recombinatorial mechanisms might also contribute to a fully blown bovine preimmune repertoire. The relative importance of V(D)J recombination for the generation of the preimmune repertoire in ruminants is currently controversial [14,18]. In late fetal and neonatal sheep, however, the repertoire is expanded by somatic hypermutation in the ileal Peyer's patch [12,13].

Surrogate light chain (SLC) is needed to expand the H^+^L^- ^cell population in species in which heavy and light chain genes are sequentially arranged. This assures that sufficient number of cells productively rearrange both loci [34]. The expression of SLC genes in the bovine fetal tissues (figure 3) confirms their functionality. The data presented in this paper does not permit further conclusions on the role of SLC genes in cattle. Nevertheless, analyses of serial sections by immunohistochemistry have revealed specific sites in the bovine fetus where there are no light chain positive cells but which still contain heavy chain positive cells (Ekman and Iivanainen, unpublished).

## Conclusion

This study describes the bovine assortment of immunoglobulin and surrogate light chain genes based on Btau_3.1. A large fraction of the potentially functional variable genes belong to subgroups that are shared between cattle and sheep but not found in man or in mouse. The number of functional light chain variable genes in Btau_3.1 is moderate in comparison with the corresponding number in the human or mouse genomes. The new data on the immunoglobulin light chain genes provides novel insight on the humoral immune system of ruminants and should facilitate the development of vaccines and other therapeutic tools against cattle specific infectious diseases.

## Methods

### Gene identification and annotation

An iterative blast search against the bovine genomic sequence database was performed via Ensembl genome browser [35]. The initial query sequences were bovine light chain variable gene encoded cDNAs with frequent matches in the dbEST database at the National Center for Biotechnology Information [36]. Genome-wide annotation evidence based on Swiss-Prot, TrEMBL and various other databases at GenBank, EMBL and DDBJ were provided by The Wellcome Trust Sanger Institute [37] and by the Bovine Genome Database [38]. Annotation of the genomic sequence and its comparison against the various evidence entries was carried out using Apollo [39], Otterlace [40] and blast [41].

### Functional and phylogenetic analyses of genes

Sequence extractions were done in the European Molecular Biology Open Software Suite [42]. The extracted genes were further analyzed using the following criteria: (a) an uninterrupted open reading frame, (b) consensus splice sites at exon/intron boundaries, (c) the presence of four conserved framework residues C23, W41, L89 and C104 for the variable and constant genes, and F/W-G-X-G motif for the joining genes [21], and (d) a likely functional recombination signal sequence. In functional recombination assays, the spacer length and three outmost nucleotides of the heptamer have been shown to be the most critical parameters for efficient recombination [43].

Multiple alignments of genomic sequences corresponding to regions spanning from FR1 up to but excluding CDR3 [21] were performed using a global alignment strategy in the MAFFT package, version 6.603b [44]. Evolutionary distances were computed and phylogenetic trees constructed in PHYLIP, version 3.67 [45] using the F84 model for nucleotide substitution and neighbor joining algorithm, respectively. The reliability of the tree topologies were evaluated using the bootstrap test (n = 1000) in PHYLIP. The consensus tree was calculated using majority rule in the Consense consensus tree program in PHYLIP.

Since the complete gene pool is not available, *ad hoc *gene names are used in this paper. The variable gene families or subgroups identified in cattle [11] and in sheep [12-15] are used where the phylogenetic analyses indicate a close relationship. Furthermore, nucleotide sequence identity matrix for the gene region corresponding to FR1–FR3 (e.g., amino acids 1 to 104 in the IMGT numbering system [21]) was calculated from globally aligned sequences using the BioEdit Sequence Alignment Editor v. 7.0.9 [46]. Truncated or incomplete genes *IGVL59*, *IGLV61*, *IGLV62 *and *IGLV63 *were excluded from the initial alignment. They were subsequently assigned to the respective subgroups by phylogenetic analysis in PHYLIP, based on alignments using the local alignment strategy in the MAFFT package (Additional file 1).

### Cloning and expression analysis of the surrogate light chain genes

Bovine fetal material was obtained from a local slaughterhouse. The use of animal tissues was approved by the local animal welfare authorities. Total RNA was isolated from muscle, thymus, liver, spleen, lymph node and bone marrow of fetuses at 135, 175, 190, 210 and 230 days of gestational age [47]. 50 – 400 mg of frozen tissue was crushed with a mortar, suspended in Eurozol RNA extraction reagent (Euroclone) and homogenized using Polytron PT1200 homogenizer (Kinematica AB) with a 5 mm cutter. The extraction procedure was carried out according to manufacturer's instructions. RNA was further purified by precipitating with 2.5 M LiCl (Sigma) and dissolved in water. Prior to reverse transcription RNA was treated with RQ1 DNAse (Promega) to remove possible genomic contamination. In the reverse transcription reaction 20 pmol of oligo(dT) primer was added to 1 μg of total RNA, and RevertAid M-MuLV reverse transcriptase (Fermentas) was used according to manufacturer's instructions. RiboLock ribonuclease inhibitor (Fermentas) was added to the reaction.

For the amplification and cloning of the full length cDNAs, the following primers were used: VPREB1-f2 and VPREB1-r1, VPREB3-fw1, VPREB3-fw2 and VPREB3-rev1, L5-f1b and L5-r3a (table 2). Purified PCR fragments were ligated to a pSTBlue-1 vector (Novagen). For each cDNA, several clones were sequenced on ABI3130 XL 16-capillary sequencer at the DNA-sequencing core facility at the University of Helsinki using fluorescently labeled BigDye™ dideoxynucleotides. To confirm suspected polymorphisms in the *VPREB1, VPREB3 *and *IGLL1 *genes, a selection of cDNA clones from lymph node and bone marrow, and PCR products from genomic DNA were sequenced.

**Table 2:** Gene specific primers used in this study

**Primer**		**Sequence**
***VPREB1***		

VPREB1-f2	C, E	5'-catgtcctgggccctcgt-3'

VPREB1-r1	C, E	5'-gcccagcctccttgtccac-3'

***VPREB3***		

VPREB3-fw1	C, E	5'-tgtgtggaggtcccgaag-3'

VPREB3-fw2	C	5'-cgcagaacagcggactcct-3'

VPREB3-rev1	C, E	5'-aggtcaggagtagaagtgg-3'

***IGLL1***		

L5-f1b	C	5'-ccagcgcgtctgcccaag-3'

L5-f2c	E	5'-tgctggctgggcgtctgg-3'

L5-r3a	C, E	5'-agaagggacgtaggggaccat-3'

***GAPDH***		

QGAPDfw	E	5'-ctgacctgccgcctggag-3'

QGAPDrev	E	5'-aagagtgagtgtcgctgttgaag-3'

The use of each primer is indicated: C (cloning of the full length cDNA), E (RT-PCR expression analysis).

The expression of *VPREB1*, *VPREB3 *and *IGLL1 *surrogate light chain genes was confirmed by RT-PCR using the following RNA preparations (age in gestational days): bone marrow (135d, 175d, 190d, 210d, 230d), liver (135d, 175d, 190d, 210d, 230d), lymph node (190d, 210d, 230d), muscle (135d, 190d, 210d, 230d), spleen (135d, 175d, 190d, 210d, 230d), and thymus (135d, 175d, 190d, 210d, 230d). Expression of the housekeeping gene *GAPDH *was used to monitor the variation in RNA quality and quantity. *GAPDH *specific control RT-PCRs without reverse transcriptase did not yield any products (not shown). For primers, see table 2.

## Abbreviations

H: heavy chain; L: light chain; FR: framework region; CDR: complementarity determining region; RSS: recombination signal sequence; IGLV: immunoglobulin lambda variable; IGLJ: immunoglobulin lambda joining; IGLC: immunoglobulin lambda constant; IGKV: immunoglobulin kappa variable; IGKJ: immunoglobulin kappa joining; IGKC: immunoglobulin kappa constant; VPREB: pre-B lymphocyte gene; IGLL: immunoglobulin lambda-like polypeptide; SLC: surrogate light chain; RAG: recombination activating gene; GAPDH: glyceraldehyde phosphate dehydrogenase; kde: kappa deleting element; RS: recombination sequence.

## Authors' contributions

AI conceived the study, analyzed and annotated the sequences, participated in the wet lab work and wrote the paper. AE and JL participitated in the wet lab work. MN participitated in the phylogenetic analyses. All authors contributed to and approved the final manuscript.

## Supplementary Material

Additional file 1 **Table S1 – Immunoglobulin λ genes in Btau_3.1**. F = functional, FL = fragmented locus, ORF = open reading frame, P = pseudogene, T = truncated pseudogene. Variations in the RSS heptamer motif and spacer length that likely prevent recombination are in bold and underlined. Scaffold coordinates are used. Click here for file

Additional file 2 **Table S2- Sequence identity matrix of ruminant immunoglobulin λ variable genes**. Sequences corresponding to the V region but excluding CRD3 were aligned and a sequence identity matrix was calculated as described in the methods. Ovine genomic sequences 1.2, 1.3, 2.1, 2.2, 3, 3.1, 4.1, 4.2, 5.1, 5.2, 5.3, 5.4, 6.1, 6.2, 8.0, 9, 10, 12.1, 12.2, 16.1, 16.2, 17, 18, 26.1, 26.2, 26.3 (AF040900–AF040924, M60441) are from Reynaud *et al*. [13]. Ovine sequences 6a, 6b, 6c and 6d (AF038145–AF038148) are derived from cDNA [14]. Identities ≥ 80% are in bold. Subgroups are bounded and colored light green. Diagonal cells are marked as ID. Click here for file

Additional file 3 **Table S3 – Immunoglobulin κ genes in Btau_3.1**. F = functional, FL = fragmented locus, ORF = open reading frame, P = pseudogene. Variations in the RSS heptamer motif that likely prevent recombination are in bold and underlined. Scaffold coordinates are used. Click here for file

Additional file 4 **Table S4 – Sequence identity matrix of ruminant immunoglobulin κ variable genes**. Sequences corresponding to the V region but excluding CRD3 were aligned and a sequence identity matrix was calculated as described in the methods. The ovine sequences 1, 2.1, 2.2, 2.3, 3 and 4 (AF038133–AF038138) are derived from cDNA [14]. Identities ≥ 80% are in bold. Subgroups are bounded and colored light green. Diagonal cells are marked as ID. Click here for file

Additional file 5 **Table S5 – Single nucleotide differences in VPREB1, VPREB3 and IGLL1**. cDNA clones or PCR products from genomic DNA were sequenced. The sequences were compared against the Btau_3.1 minus strand of Chr17.003.88 scaffold reference sequence. Both alleles are indicated whenever possible. Click here for file

Additional file 6 **Table S6 – V and J region amino acid sequences of the apparently functional bovine immunoglogulin light chains**. Dots indicate gaps that were introduced during the alignment. White spaces separate consecutive FRs and CDRs. Click here for file
