# Supplementary material for: Bos taurus genome sequence reveals the assortment of immunoglobulin and surrogate light chain genes in domestic cattle
Source: BMC Immunol. 2009 Apr 30;10:22. doi: 10.1186/1471-2172-10-22 (PMC2686674; doi:10.1186/1471-2172-10-22)
Supplement: Additional file 6 — Table S6 – V and J region amino acid sequences of the apparently functional bovine immunoglogulin light chains. Dots indicate gaps that were introduced during the alignment. White spaces separate consecutive FRs and CDRs. [file 1471-2172-10-22-S6.pdf]

```
        |------------FR1-----------|----CDR1----|-------FR2-------|---CDR2---|------------------FR3-----------------|---CDR3---|
Lambda variable genes, subgroup 1
IGLV2    QAVLTQPSS.VSGSLGQRVSITCSGS SNNIGSYG.... VGWYQQVPGSGLRTIIY GSS....... SRPSGVP.DRFSGSK..SGNTATLTISSLQAEDEADYFC ATVDYSSSTV
IGLV6    QDVLTQPSS.VSGSLGQNVSITCSGS SSNVGYANY... VSWHQQKQGSAPRTLIY GAT....... SRASGVP.DQFSGSK..SGNTATLTISSLQPEDEADYYC SSYDSSSNIG
IGLV8    QAVLTQPPS.VSGSLGQTVTISCTGS SNNIGILG.... VSWYQQIPGSAPRTLIY NSN....... KRPSGVP.DRFSGTK..SGNTGTLTIASLQAEDEADYYC ASADLSLTSP
IGLV23   QAVLTQPPS.VSGSLGQRVTISCTGS SSNIGGGNY... VGWYQQIPGSAPKTLIY RST....... SRPSGVP.DRFSGSR..SGNTATLTISSLQAEDEADYYC ATYESSSYHG
IGLV25   QAVLTQPSS.VSGSLGQRVSITCSGS SNNIGRYG.... VGWYQQVPGSGLRTIIY GSS....... SRPSGVP.DRFSGSK..SGNTATLTISSLQAEDEADYFC VAYDSSSSIV
IGLV27   QAVLTQPSS.VSGSLGQRVSITCSGS SSNVGYGNY... VSWFQEIPGSAPRTLIY GDT....... SRASGVP.DRFSGSR..SGNTATLTISSLQAEDEADYFC ASYQSGNT
IGLV28   QAVLTQPSS.VSGSLGQRVSITCSGS SSNVGTGNY... VSWFQQIPGSAPRTLIY GAT....... SRASGVP.DRFSGSR..SGNTATLTISSLQAEDEADYFC ASYQSGNT
IGLV30   QAVLTQPSS.VSGSLGQRVSITCSGS SSNVGNGY.... VSWYQLIPGSAPRTLIY GDT....... SRASGVP.DRFSGSR..SGNTATLTISSLQAEDEADYFC ASAEDSSSNA
IGLV33   QAVLTQPSS.VSGSLGQRVSITCSGS SSNVGTGNY... VSWFQQIPGSAPRTLIY GAT....... SRASGVP.DRFSGSR..SGNTATLTISSLQAEDEADYFC ASYQSGNT
IGLV35   QAVLTQPSS.VSGSLGQRVSITCSGS SNNIGRYG.... VGWYQQVPGSGLRTIIY GSS....... SRPSGVP.DRFSGSK..SGNTATLTISSLQAEDEADYFC ATGDYSSSTV
IGLV37   QAVLTQPSS.VSGSLGQRVSITCSGS SSNVGYGNY... VSWFQEIPGSAPRTLIY GDT....... SRASGVP.DRFSGSR..SGNTATLTISSLQAEDEADYFC ASYQSGNT
IGLV39   QAVLTQPSS.VSGSLGQRVSITCSGS SNNIGSYG.... VGWYQQVPGSGLRTIIY GSS....... SRPSGVP.DRFSGSK..SGNTATLTISSLQAEDEADYFC AAGDSSSSTG
IGLV43   QAVLTQPSS.VSGSLGQRVSITCSGS SSNVGYGNY... VSWFQQIPGSAPRMLIY GAT....... SRASGVP.DRFSGSR..SGNTATLTISSLQAEDEADYFC ASPDSSSSG
IGLV45   QAVLTQPPS.VSGSLGQRVTISCTGS SSNIGGGNC... VGWYQQIPGSAPKTLIY RST....... SRPSGVP.DRFSGSR..SGNTATLTISSLQAEDEADYYC ATYESSSYHG
IGLV49   QAVLTQPSS.VSGSLGQRVSITCSGS SSNVGLGNY... VSWFQQIPGSAPRTLIY GAT....... SRASGVP.DRFSGSR..SGNTATLTISSLQAEDEADYFC ASPDSSSSS
IGLV55   QAVLTQPSS.VSGSLGQRVSITCSGS SSNVGRGNY... VNWFQQIPGSAPRTLIY GAT....... SRASGVP.DRFSGSR..SGNTATLTISSLQAEDEADYFC AAGDSSSSTG

Lambda variable genes, subgroup 2
IGLV10   QSGLTQPSS.VSGNLGQTVTITSCAGT SSYVGSYNG... VGWYQQLPGSAPKTLIY NVS....... KRPSGIP.DRFSGSK..SGNTATLTVSGLQAEDEADYYC SSYKSGGSV
IGLV13   QSGLTQPSS.VSGNLGQTVTISCAGT SSDVGAYNG... VGWYQQLPGSAPKTLIY NLN....... KRSSGIP.ARFSGSK..SGNTATLTISGLQAEDEADYYC SSYKSGGSV

Lambda variable genes, subgroup 6
IGLV14   SSQLTQPPA.VSVSLGQTASITCQGD DLESYY...... AHWYQQKPSQAPVLVIY ESS....... ERPSGIP.DRFSGSS..SGNTATLTISGAQTEDEADYYC QSYDSSGDP
IGLV15   SYELTQPTS.VSVALGQTAKITCSGD LLDEQY...... TQWYQQKPGQGPVRVIY KDS....... ERPSGIS.DRFSGSS..SGKTATLTISGAQTEDEADYYC QSADSSDNP
IGLV16   SYELTQLTS.VSVALGQTAKITCSGE LLDEQY...... TQWYQQKPGQAPKLVIY KDS....... KRRSGIP.DQFSGSS..SGKTAILTISGVRAEDEADYYC LSWDSGSYNV
IGLV17   SSQLTQPPA.VSVSLGQTASITCQGD DLELLS...... AHWYQQKPGQAPVLVIY ADD....... NLASGIP.DRFSGSK..SDTTATLTIRGAQAEDEADYYC QSADISGV

Lambda variable genes, subgroup 7
IGLV21   QPVLTQPVT.VSASLGASARLSCTLS SGYNVSNYS... IYWYQQKAGNPLRYLLR FKSDSDK... HQGSGVP.SRFSGSKDASTNAGLLLISGLQPEDEADYYC AVWHGDTNA

Lambda variable genes, subgroup 8
IGLV40   QTVI.QEPS.LSVSPGGTVTLTCGLS SGSVTTYNE... PSWYRETPGQAPRNVIY NTN....... TPRTGVP.DRFSASI..SGNKVTLTITGAQPEDEADYHC LLYQGSDSYG
IGLV46   QTVI.QEPS.LSVSPGGTVTLTCGLS SGSVTTYNE... PSWYRETPGQAPRNVIY NTN....... TPRTGVP.DRFSASI..SGNKVTLTITGAQPEDEADYLC LLYQGSDSYG

Kappa variable genes, subgroup 2
IGKV8    DVVLTQTPLSLSVIPGETVSISCKST QSLKYSGKTY.. LRWLQHKPGQSPQSLIY QVS....... NRYTGVP.DRFTGSG..SETDFTLTISSVQAEDAGVYYC VQETHDPR
IGKV10   DVVLTQTPLSLSVIPGETVSISCKST QSLKYSDGKTY. LRWVQHKPGQSPQGVIY QVS....... NRNTGVP.DRFTGSG..SETDFTLTISSVQAEDAGVYYC FQGTYEPP
IGKV11   DVVLTQTPLSLSVIPGETVSISCKST QSLKYSGKTY.. LRWLQHKPGQSPQSLIY QVS....... NRYTGVP.DRFTGSG..SETDFTLTISSVQAEDAGVYYC VQETHDPR
IGKV13   DVVLTQTPLSLSVIPGETVSISCKST QSLKYSDGKTY. LRWVQHKPGQSPQGVIY QVS....... NRNTGVP.DRFTGSG..SETDFTLTISSVQAEDAGVYYC FQGTYEPP
IGKV14   DVVLTQTPLSLSVIPGETVSISCKST QSLKYSGKTY.. LRWLQHKPGQSPQSLIY QVS....... NRYTGVP.DRFTGSG..SETDFTLTISSVQAEDAGVYYC VQETHDPR
IGKV19   DVVLTQTPLSLSVIPGETVTISCKST QSLKYSDGKTY. LQWFQHKPGQSPRLLIY QIS....... NRYTGVP.DRFTGSG..SETDFTLTISSVQAEDAGVYYC LQRSYAPR
IGKV21   DVVLTQTPLSLSIIPGEMASISCKSS QSLVHSDGKTY. LNWIQYKPGQSPQGLIY QVS....... NRYSGVS.DRFTGSG..SGTDFTLTISRVQAEDAGVYYC YQGTEDPP

Kappa variable genes, subgroup 4
IGKV23   DIQVTQSPSYLSASLGDRVSITCQAN QSVSHY...... LNWYQQKPGEAPKLLIY YAT....... SRYTRVP.SRFSGSG..SGTDFTLTISSLEADDAANYYC QQDYSTPP

Lambda joining genes
IGLJ2    DLFGGGTRVTVL
IGLJ3    AVFGSGTTLTVL

Kappa joining genes
IGKJ1    NTFGQGTKVEIK
IGKJ3    ITFGGGTKVEIN
```
